# Retrospective analysis of quantitative electroencephalography changes in a dissimulating patient after dying by suicide: A single case report

**DOI:** 10.3389/fpsyt.2023.1002215

**Published:** 2023-03-15

**Authors:** Tomáš Rakús, Katarína Hubčíková, Lucia Bruncvik, Zuzana Petrášová, Martin Brunovsky

**Affiliations:** ^1^Department of Neuropsychiatry, Philippe Pinel Psychiatric Hospital, Slovak Medical University in Bratislava, Pezinok, Slovakia; ^2^Third Faculty of Medicine, Charles University in Prague, Prague, Czechia; ^3^Landesklinikum Hainburg, Hainburg an der Donau, Austria; ^4^Department of Neurophysiology and EEG, National Institute of Mental Health, Klecany, Czechia

**Keywords:** suicidality, depressive disorder, theta cordance, quantitative electroencephalography, dissimulation

## Abstract

We present the case of a 49-year-old man who was diagnosed with depressive disorder, with the first episode having a strong reactive factor. He was involuntarily admitted to a psychiatric hospital after a failed attempt at taking his own life, where he responded to psychotherapy and antidepressant therapy, as evidenced by a >60% reduction in his MADRS total score. He was discharged after 10 days of treatment, denied having suicidal ideations, and was motivated to follow the recommended outpatient care. The risk for suicide during hospitalization was also assessed using suicide risk assessment tools and psychological assessments, including projective tests. The patient underwent a follow-up examination with an outpatient psychiatrist on the 7th day after discharge, during which the suicide risk assessment tool was administered. The results indicated no acute suicide risk or worsening of depressive symptoms. On the 10th day after discharge, the patient took his own life by jumping out of the window of his flat. We believe that the patient had dissimulated his symptoms and possessed suicidal ideations, which were not detected despite repeated examinations specifically designed to assess suicidality and depression symptoms. We retrospectively analyzed his quantitative electroencephalography (QEEG) records to evaluate the change in prefrontal theta cordance as a potentially promising biomarker of suicidality, given the inconclusive results of studies published to date. An increase in prefrontal theta cordance value was found after the first week of antidepressant therapy and psychotherapy in contrast to the expected decrease due to the fading of depressive symptoms. As demonstrated by the provided case study, we hypothesized that prefrontal theta cordance may be an EEG indicator of a higher risk of non-responsive depression and suicidality despite therapeutic improvement.

## Introduction

Unless the symptoms of a serious psychiatric illness are clearly expressed, predicting the risk of suicide in a dissimulating patient is a serious problem. Clinical assessment of suicide risk can be significantly improved, especially in the case of less experienced therapists, by using standardized psychometric scales for suicide risk assessment, such as the Columbia Suicide Severity Rating Scale (C-SSRS) (1) or by using projective methods based on the assumption that suicidal intentions can be mirrored in response to ambiguous stimuli, whether at a conscious level (e.g., dissimulation) or on an unconscious level (e.g., presuicidal development). According to the review by Kumar et al. (2) the Rorschach test is the most commonly used projective test in the assessment of suicidality. In addition, even with antidepressant treatment, it must be taken into consideration that the patient's symptoms may appear mild but that any improvement is in fact only presumed and may be based on the patient's final decision to take their own life (or at least attempt it again). To date, there are no clinically useful objective markers to assess suicide risk in patients with depressive disorders or other mental health conditions. According to a recent review article (3), dysfunction of the frontotemporal networks with a volume reduction in the gray and white matter in the prefrontal cortex, the anterior cingulate, and the upper anterior temporal gyrus of the brain of individuals with suicidal ideation across psychiatric diagnoses (major depressive disorder, bipolar disorder, psychotic disorders, and borderline personality disorder) was pointed out using functional neuroimaging (positron emission tomography and functional magnetic resonance) and structural neuroimaging methods (computed tomography, magnetic resonance imaging, and diffusion tensor imaging). These findings corroborate the hypothesis that executive dysfunction has a direct impact on emotional regulation in individuals with suicidal ideation, where emotion-regulation strategies are disrupted and maladaptive procedures with reduced ability to cope with emotional distress are used instead (4). Advanced electrophysiological methods such as quantitative electroencephalography (QEEG) have been investigated in the field of suicidality. QEEG is a cost-effective method that can be easily replicated without the need for administering radioactive substances or generating a strong magnetic field. In studies on suicidality, frontal QEEG changes have shown promise (5–9), especially changes in theta activity [(4–8) Hz], which are closely related to the anterior cingulate activity (10). These changes can be measured by assessing absolute/relative power, (a) symmetry [the ratio of power in each band between symmetrical pairs of electrodes in the left and right hemispheres (11)], coherence (the degree of synchronization between two channels), and cordance [which allows assessment of the integrity of afferent inputs in one brain area (12)] or using Low-Resolution Brain Electromagnetic Tomography (LORETA). Cordance combines complementary information from the absolute and relative power of EEG spectra to provide values that correlate more strongly with regional cerebral perfusion (as measured using ${\text{H}}_{2}^{15}$O positron emission tomography) than QEEG measures alone (13). In addition, prefrontal cordance is a new and promising method for predicting antidepressant efficacy using baseline and/or 1-week changes in QEEG (7). Changes in QEEG theta cordance in the brain of individuals with suicidal ideation have been confirmed by several studies. Higher theta cordance is associated with suicidal ideation (8), and post-mortem analysis of an individual who took her own life, having previously displayed disguised clinical symptoms (despite psychometric scales), showed higher theta cordance 24 h before burning herself to death (5). In contrast, a decrease in theta cordance is associated with suicidality, a fact which was confirmed by Hunter et al. (14). Researchers have also examined the asymmetry of the power in individual EEG frequency bands, indicating that higher asymmetry of the combined theta and alpha power is correlated with worsening of depressive symptoms in the study by Ioniflescu et al. (6), whereas a lower frotal alpha asymmetry was associated with more suicidal ideation in the study by Roh et al. (15). The conflicting findings may be due to differences in how suicide risk was assessed, (ranging from a lifetime history of suicidal attempts to having a positive score on use of psychometric suicide scales), as well as variations in pre-test medication that could affect EEG data availability (16).

## Case

The presented case study focuses on the impact of the suicidal behavior of a 49-year-old man after he was discharged from a psychiatric hospital despite not being deemed at risk for suicide. This patient was admitted to a psychiatric hospital after a failed attempt to take his own life (he sent a text message to his wife that he was going to jump off a bridge, from where he was rescued by the police). The psychiatric examination revealed depressed mood, anhedonia, increased fatigue, loss of self-confidence, and self-blame (but no delusional depth), mainly regarding the feeling of failure as a husband in a disharmonious marriage, persistent suicidal ideation, concentration problems, terminal insomnia, and loss of appetite. Most of the symptoms had been present for 3 weeks before the attempt at taking his life. No features or history (including an objective history obtained from the daughter) of bipolar affective disorder, recurrent depressive disorder, or personality disorder were present. In addition, a CT scan of the brain and laboratory tests, including thyroid hormones, alcoholemia, and urine toxicology, were completely negative. The patient was diagnosed with first-episode depressive disorder with a significant contributing factor identified as marital disharmony. On the first day after admission, the patient provided informed consent for QEEG assessments, which were part of a larger study on the correlation between QEEG measurements and depressive symptoms and suicidality. The study was approved by an ethics committee at the Philippe Pinela Psychiatric Hospital, Pezinok, Slovakia, in accordance with the 1995 Declaration of Helsinki (as revised in Edinburgh in 2000). The patient's written informed consent also included consent for potentially identifiable images or data to be published in this article. The QEEG assessments were conducted on the morning of the 2nd day of hospitalization (when the patient was not yet medicated and had no history of medication for at least 6 months prior to the suicide attempt) and on the 9th day of hospitalization (when the patient was medicated with sertraline). He responded to psychotherapy with a >60% reduction in the total score of the Montgomery-Asberg Depression Rating Scale (MADRS) (17) and was prescribed an antidepressant (sertraline 100 mg per day). He was discharged after 10 days of treatment. He denied having any suicidal ideation and was motivated to participate in marriage counseling. Suicidal risk during hospitalization was also assessed using the C-SSRS and the Rorschach test, including suicidal assessment according to the Piotrowski method of Rorschach [interpreted by Kendra (18)]—both found no acute suicidal risk. The psychological examination (including ROR) also pointed out that his suicidal behavior was demonstrative, which served its purpose and initiated communication between the spouses about their problems. The patient displayed neurotic tendencies, had a fear of aggressive behavior, and had difficulty expressing himself assertively or engaging in any interpersonal conflict. The patient was re-evaluated using C-SSRS and MADRS, as well as by an outpatient psychiatrist 7 days after discharge, and the patient showed no signs of acute suicidal risk or worsening of depressive symptoms. The dynamics of the results (absolute values) of the used psychometric rating tools are summarized in Table 1.

**Table 1:** An overview of the dynamics of the results (absolute values) of the used psychometric rating tools.

**Scale/protocol for suicidal risk assessment**	**Day after patient's failed attempt to die by suicide**		
	**1st**	**7th**	**14th**
MADRS	54	21	17
C-SSRS (lifetime version)	Suicidal ideation/intensity of suicidal ideation 5/20 Suicidal behavior/actual lethality/potential lethality Actual attempt/0/2		
C-SSRS (since the last visit)		0	0
ROR (Piotrowski suicidal protocol)		+1	

MADRS, Montgomery-Asberg Depression Rating Scale; C-SSRS, Columbia–Suicide Severity Rating Scale; ROR, Rorschach.

**Figure 1:**
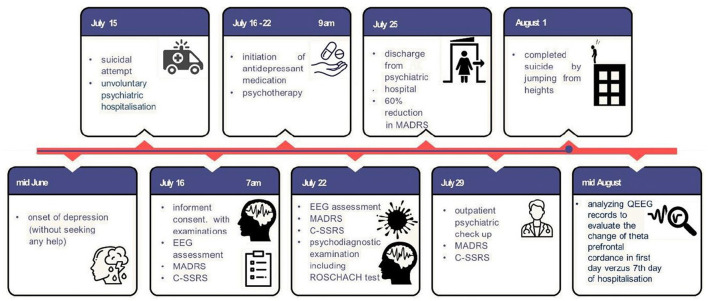
Timeline of presented case report. EEG, Electroencephalography; MADRS, Montgomery–Asberg Depression Rating Scale; C-SSRS, Columbia–Suicide Severity Rating Scale; QEEG, Quantitative Electroencephalography.

**Figure 2:**
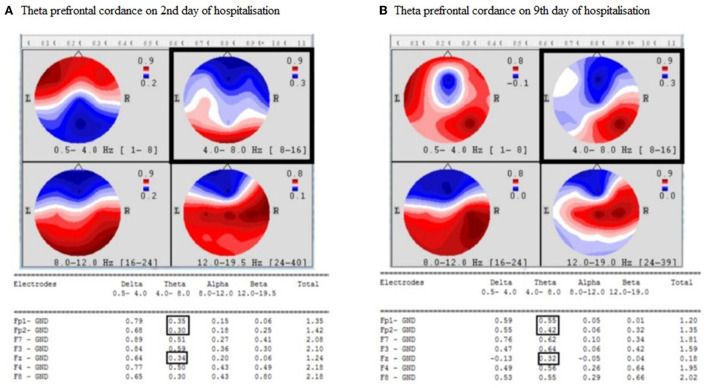
**(A, B)** Increase of theta prefrontal cordance in presented patient. Hz, Hertz; L, Left; R, Right, Electrodes; Fp, Frontopolar; F, Frontal; Fz, Midline frontal; GND, Ground.

On the 10th day after discharge, the patient committed suicide by jumping out of the window of his flat. We retrospectively analyzed his QEEG recordings to evaluate the change in theta prefrontal cordance. The timeline of the presented case study is presented in Figure 1. We used the same algorithm as Leuchter et al. (13, 19). The theta prefrontal cordance value was 0.33 before the start of treatment with sertraline and psychotherapy and 0.43 after 7 days of therapy. The exact values of theta cordance and their graphical brain representations are presented in Figure 2. Written informed consent for the publication of any potentially identifiable images or data included in this article was also obtained from the deceased patient's next of kin.

## Discussion

The patient was assessed as a responder based on the reduction in MADRS total score following antidepressant therapy and outgoing psychotherapy. Nevertheless, he took his own life on the 10th day after his discharge and the 3rd day after his outpatient psychiatry check. Considering his previous clinical symptoms, we suspect that the patient dissimulated the severity of his depressive symptoms and overestimated his marital problems, which may have led to his suicide. In addition, we did not observe a reduction in the theta prefrontal cordance, which is believed to be an early biological predictor of antidepressant response (20, 21). However, we did observe an increase in theta prefrontal cordance 10 days before the patient suicide, which may be an electrophysiological marker of the patient's suicidality (5, 8). There may be an alternative explanation for the observed results. His suicide could also have been an impulsive act, especially given the nature of his suicide, but this assumption is inconsistent with the results of the psychological examination, which included projective methods and was conducted by an experienced clinical psychologist. If the patient was misdiagnosed and it was a repeated depressive reaction to returning to a stressful environment, then it is appropriate to consider whether the increase in theta cordance may have represented an ongoing suicidal risk in a vulnerable patient rather than dissimulated depression. We may also hypothesize that the patient was mostly a placebo responder, which is characterized by a significant early increase in prefrontal cordance values that are not observed in antidepressant or placebo non-responders (19). This placebo response could be transient and could explain the discrepancy between the decrease in depressive symptoms and the sudden onset of a depression relapse.

## Conclusion

We suggest that increased theta prefrontal cordance may be an objective marker for depressive symptoms or even suicidality that are unaffected by dissimulation, unlike psychometric rating scales or projective psychological tests. Further research in this area is needed, as objective measures of this nature could prove to be clinically useful, especially for patients at a high risk of suicide who struggle to accurately articulate their symptoms.

## Data availability statement

The raw data supporting the conclusions of this article will be made available by the authors, without undue reservation.

## Ethics statement

The studies involving human participants were reviewed and approved by Ethics Committee of the Psychiatric Hospital of Philipp Pinel in Pezinok. The patients/participants provided their written informed consent to participate in this study. Written informed consent for the publication of any potentially identifiable images or data included in this article was obtained also from deceased patient's next of kin.

## Author contributions

TR and KH participated mainly in the design of the study, literature searches and analyses, statistical analyses, and interpretation of data. ZP and LB participated mainly in writing the manuscript. MB supervised the study. All authors contributed to the article and approved the submitted version.
